# Extraction Processes with Several Solvents on Total Bioactive Compounds in Different Organs of Three Medicinal Plants

**DOI:** 10.3390/molecules25204672

**Published:** 2020-10-13

**Authors:** Nour El Houda Lezoul, Mohamed Belkadi, Fariborz Habibi, Fabián Guillén

**Affiliations:** 1 Département de Génie Chimique, Faculté de Chimie, Université des Sciences et de la Technologie d’Oran Mohamed Boudiaf, Laboratoire de Synthèse Organique, Physico-Chimie, Biomolécules et Environement (L.S.P.B.E), USTO-MB, BP 1505, El M’naouer, Oran 31000, Algeria; nourelhouda.lezoul@univ-usto.dz (N.E.H.L.); belkadi101@yahoo.fr (M.B.); 2 Department of Horticultural Science, School of Agriculture, Shiraz University, P.O. Box 7144165186, Shiraz 99907, Iran; fariborz_h659@yahoo.com; 3 Department of Food Technology, University Miguel Hernández, Ctra. Beniel km. 3.2, Orihuela, 03312 Alicante, Spain

**Keywords:** maceration, decoction, total polyphenol content, flavonoids, antioxidant activity

## Abstract

The extraction of secondary metabolites by water, MeOH:water (8:2) containing NaF, methanol, ethanol and acetone (all of them diluted (7:3) in water)from the different parts (leaves, flowers, stems and roots) of *Passiflora caerulea* L., *Physalis peruviana* L. and *Solanum muricatum* Aiton via decoction and maceration methods was studied. The highest extraction yields were recorded by methanol for decoction and acetone for maceration. The total polyphenol content (TPC) obtained by decoction had the highest TPC contents, and MeOH containing NaF was the best solvent for the extraction of TPC. Maceration was suitable for flavonoid extractions, with ethanol and acetone being the best solvents. In general, the highest levels of TPC and flavonoids were obtained from *Passiflora* leaves regardless of the solvent or extraction method applied. Furthermore, the roots of *Physalis* and *Solanum* showed important levels of these compounds in consonance with the total antioxidant activity (TAA) evaluated in the different organs of the plant in the three species. In this study, the solvents and extraction methods applied were tools that determined significantly the level of extraction of bioactive compounds, showing a different impact on plant organs for each medicinal species studied.

## 1. Introduction

Polyphenols are natural compounds that are widely found in plants and their importance continues to grow, in particular because of their impact on organoleptic and health benefits [1,2]. Their role as natural antioxidants is useful in the prevention and treatment of cancer, inflammation and cardiovascular diseases [2]. Indeed, in the extraction of active ingredients with high added value from plants, especially polyphenols, the extraction method applied is a very important step in the isolation, as well as in the identification, of phenolic compounds. As a result, many authors have studied the influence of different extraction methods on the extraction yields of phenolic compounds in plant sources [3]. Among others things, the solubility of the phenolic compounds is affected by the polarity of the solvent used. Further, the determination of phenolic compounds from plant materials is influenced by the extraction technique, the extraction time and the temperature. Therefore, it is difficult to develop an extraction process suitable for the extraction of all the phenolic compounds of plants [3,4,5].

Despite several disadvantages, liquid-liquid and solid-liquid extraction are still the most commonly used extraction procedures for phenolic acids and flavonoids. For many years, the conventional techniques have been widely accepted, mainly because of their ease of use, efficiency, and wide-ranging applicability [6]. Maceration and decoction techniques are very useful methods for weakening the cell wall in vegetal tissues, but in different ways. The main difference between them regards temperature. High temperatures applied with decoction can destroy bioactive compounds, such as polyphenols, so the application of milder temperatures through a gentle maceration process would be a better option [7]. However, applying high temperatures during a decoction process can also destroy enzymes, preventing the degradation of polyphenols [8]. Some modern extraction methods, such as microwave-assisted extraction, ultrahigh pressure extraction and supercritical carbon dioxide extraction, have been utilized for the preparation of plant extracts [9]. These methods have various benefits, such as the improved penetration of solvents into plant particles, a low extraction temperature and reduced extraction time, but maceration is simple, more convenient and less costly in terms of instrumentation [10]. Therefore, it is more applicable in small and medium enterprises (SMEs) and in developing countries [11]. The use of medicinal plants is the first choice of treatment for areas where access to appropriate care is difficult, while traditional herbal medicines provided by traditional healers are fast and available at all times when needed. In this sense, extraction methods based on decoction and maceration are the most used generally [12].

Bioactive compounds, including flavonoids, phenols, anthocyanins, ascorbic acid, amides, alkaloids, tannins, saponins and glycosides, from different part of medicinal species play an important role in human health, due to their biological activity [13].

*Passiflora caerulea* L., better known as the ‘blue passion flower’ [14], belongs to the family Passifloraceae, which is composed of 18 genera and about 630 species [15]. *Passiflora* is a rich source of phenolic compounds, amino acid α-alanine and organic acids, including butyric, formic, oleic, linoleic, citric, malic, myristic, linolenic and palmitic acids [16]. *Passiflora* plants are used in traditional medicine in South America to treat various pathologies associated with the gastrointestinal tract [17,18], and also in the Netherlands, Spain, Italy and Poland [19]. They exhibit various pharmacological properties and possess biologically active complex compounds [20].

*Physalis peruviana* L. is a plant of the family Solanaceae and the genus *Physalis*, which has about 100 species [21]. It is commonly referred to as the gooseberry [22]. *Physalis peruviana* is a medicinal plant widely used in traditional medicine to treat diseases such as malaria, asthma, hepatitis, dermatitis, cancer and rheumatism. It has antispasmodic, diuretic, antiseptic, sedative, analgesic, antioxidant, antifungal, antibacterial, anti-inflammatory, cataract-cleansing, antidiabetic and antiparasitic properties [23,24,25,26,27,28]. It has been described as a good source of nutrients and bioactive compounds, β-carotene and vitamins A and C, and minerals including K, Mg and Cu, with moderate fibre content, phenolic compounds and low levels of calories [29,30].

*Solanum muricatum* Aiton is a horticultural crop commonly known as ‘pepino’ or ‘pear melon’, and is a member of the family Solanaceae [31]. It is a plant known for its medicinal uses [32], with fruit which are very rich in minerals (Fe, Zn, Cu, Mn, Ca and P) and vitamin C but poor in starch and sugars [33]. It has also been found that the phenolic content of the pepino fruit is much higher than that of vitamin C [34]. In addition to these nutritional aspects, *Solanum muricatum* Aiton is known for its anti-tumor, antioxidant, antidiabetic and anti-inflammatory properties [35,36].

The investigation of these plants and their derived chemical compounds offers a different source of natural products, which could be a promising area of functional ingredient study. This type of processing is very sustainable in industrial production, as it has a secured market for the final product (food and pharmaceutical industry) and cheap raw material [37]. In this sense, during the production of passion fruit, cape gooseberry or pepino, some parts of the plant are usually disposed as waste, but could provide a source of income.

In this context, the objective of our study is to determine the best technique for extracting total polyphenols and flavonoids from different parts of the aforementioned tropical plants using different extraction solvents, and to determine their antioxidant activities.

## 2. Results

### 2.1. Extraction Yield

Both extraction methods (decoction and maceration) showed significant differences (*p* < 0.05) for all species and plant parts used in this research.

#### 2.1.1. Extraction Yield by Maceration

The results of the maceration extraction yield (Table 1) show that acetone is the best extraction solvent, with an average of 20.43 ± 0.23% for *Passiflora*, 20.95 ± 0.15% for *Physalis* and 20.01 ± 0.18% for *Solanum*, followed by water, with averages of 19.53 ± 0.23%, 18.67 ± 0.14% and 18.32 ± 0.19% for *Passiflora*, *Physalis* and *Solanum*, respectively. The highest extraction in *Solanum* was found in the leaves for all solvents used (*p* < 0.05).

In general, the leaves recorded the best yield for *Passiflora* and *Solanum*, averaging 18.51 ± 0.22% and 18.79 ± 0.21%, respectively, followed by roots (18.33 ± 0.21%), flowers (16.93 ± 0.22%) and stems (16.75 ± 0.20%) for *Passiflora*. The flowers, roots and stems had average yields of 17.76 ± 0.18%, 17.04 ± 0.21% and 16.23 ± 0.19%, respectively, for *Solanum*.

On the other hand, the highest yield recorded from the averaging of *Physalis* was that of the roots (18.57 ± 0.16%), followed by stems (18.11 ± 0.16%), flowers (17.89 ± 0.17%) and leaves (17.40 ± 0.16%).

#### 2.1.2. Extraction Yield by Decoction

In contrast to the results of the yields obtained by maceration, the yields obtained by decoction when methanol was used as an extractant solution (Table 1) were the highest yields, with averages of 21.58 ± 0.24% for *Passiflora caerulea*, 20.89 ± 0.14% for *Physalis peruviana* and 21.57 ± 0.26% for *Solanum muricatum* on four samples (leaves, flowers, stems, roots), while acetone gave the lowest yield for *Passiflora*, *Physalis* and *Solanum*, with averages of 14.83 ± 0.15%, 14.39 ± 0.14% and 11.15 ± 0.23%, respectively. *Physalis peruviana* exhibited the highest extraction level from its roots when methanol was used, showing a significant difference (*p* < 0.05) in comparison with other parts of the plant, such as leaves and stem. On the other hand, *Passiflora* and *Solanum* in general yielded higher levels of extraction in both roots and leaves, with significant differences (*p* < 0.05) between these plant parts, especially for *Solanum*.

### 2.2. Total Polyphenol Content (TPC)

The data analysis showed significant differences (*p* < 0.05) in the total polyphenol content (TPC) among the plant parts (Table 2). After dosing and calculation of the TPC extracted by decoction and by maceration for the different parts of the *Passiflora* and *Physalis* plants, we can conclude that extraction by decoction gave better general results compared to solvents extracted by maceration.

The highest TPC level extracted from the different parts of *Passiflora caerulea* and *Solanum muricatum* Aiton via the decoction method with methanol in combination with sodium fluoride (MeOH NaF) was recorded in general at the leaves, followed by the roots. The total polyphenol content in these two species are given in the following descending order: leaves > roots > stems > flowers). In this sense, the TPC in the leaves of *Passiflora caerulea* and *Solanum muricatum* Aiton exhibited the highest levels for decoction (1976.95 ± 62.04 and 1637.82 ± 47.29 mg eq GA/100 g DM, respectively), followed by the roots (1466.08 ± 49.69 and 1398.69 ± 36.46 mg eq GA/100 g DM, respectively). The highest TPC contents in the different parts of *Passiflora caerulea* and *Solanum muricatum* Aiton via the maceration method were obtained with MeOH NaF from the leaves (1597.17 ± 41.45 and 1824.34 ± 27.72 mg eq GA/100 g DM, respectively). In addition, regardless of the solvent used, the total polyphenol content in the different parts of *Physalis peruviana* were obtained in the following order: roots > leaves > stems > flowers. This order was independent of the method applied (maceration or decoction), but the TPC level obtained was in general higher when the decoction method was applied.

MeOH NaF recorded the highest polyphenol content, this being significantly different (*p* < 0.05) in comparison with the other solvents for the three species. The potential for extraction of TPC via maceration by the rest of the solvents could be classified in a different order, regarding the species studied, i.e., *Passiflora* and *Solanum* (ethanolic extracts > acetonic extracts > methanolic extracts) or *Physalis* (acetonic extracts > ethanolic extracts > methanolic extracts). During decoction, methanolic and acetonic extracts showed less differences regarding the potential for extraction between them in the three species evaluated, but especially for *Passiflora* and *Solanum* the best solvent used in a decoction process was ethanol, in comparison with acetone or methanol.

### 2.3. Flavonoid Content

The flavonoid contents of the decocts and macerates of the four parts of the plant under study, obtained by the different solvents (Table 3), show that maceration is preferable for extracting flavonoids in general from the leaves and flowers of *Solanum* and for *Passiflora* leaves, which recorded the highest level, with a mean of 736.10 ± 5.38 mg eq Qu/100 g DM for MeOH NaF. On the other hand, decoction was in general the best method for an optimal flavonoid extraction from the flowers, stems and roots of *Physalis* and *Passiflora.*

There was a significant difference (*p* < 0.05) in flavonoid content among plant parts, as was expected. The great differentiation between parts of the plant appears to be related to the high levels of flavonoids in some parts of these plants, especially in the leaves and roots of the species in this study. The flavonoid content in the different parts of *Passiflora* and *Solanum* was higher in the leaves than in the rest of the parts of the plant regardless of the solvent applied to the macerated and decocted samples. In general, for *Physalis* under maceration conditions, ethanolic and acetonic extracts were the extraction solvents that recorded the highest flavonoid levels, regardless of the part of the plant, in almost all samples studied. The flavonoid levels derived from the different parts of *Physalis*, in both the macerated and decocted samples, were given in the following descending order: roots and leaves > stems > flowers (Table 3).

### 2.4. Total Antioxidant Activity

**Figure 1:**
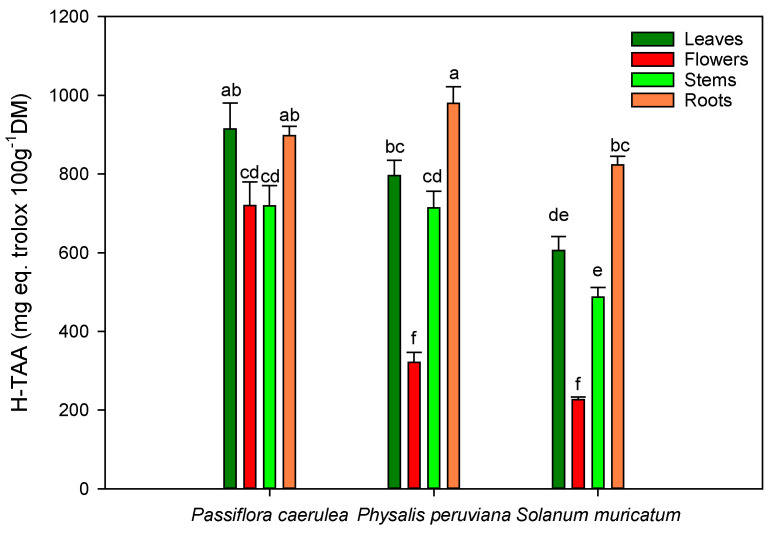
Antioxidant activity of the different parts of *Passiflora caerulea* L., *Physalis peruviana* L. and *Solanum muricatum* Aiton in the hydrophilic soluble phase. Different letters on the bars indicate significant differences at a *p* < 0.05 level of probability. Data are the mean ± standard error (SE).

**Figure 2:**
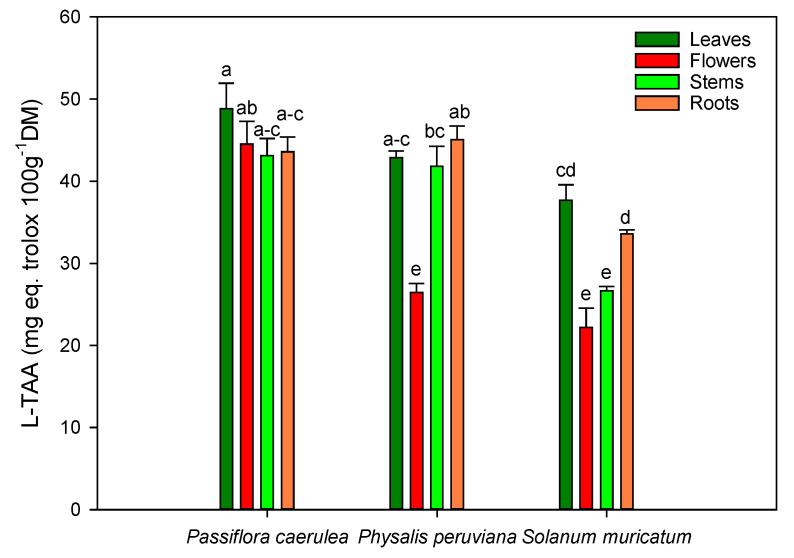
Antioxidant activity of the different parts of *Passiflora caerulea* L., *Physalis peruviana* L. and *Solanum muricatum* Aiton in the lipophilic soluble phase. Different letters on the bars indicate significant differences at a *p* < 0.05 level of probability. Data are the mean ± standard error (SE).

In our samples, total antioxidant activity (TAA) was measured in the hydrophilic (H-TAA) and lipophilic (L-TAA) fractions, shown in Figure 1 and Figure 2. The results showed that the H-TAA fractions of *Passiflora*, *Physalis* and *Solanum* obtained the highest levels of H-TAA in the leaves and roots. These levels were significantly higher (*p* < 0.05) in the roots of *Physalis* and *Solanum* (979.54 ± 42.43 and 823.62 ± 22.06 mg eq trolox/100 g DM, respectively) than in other parts of the plant (Figure 1). The leaves and flowers of *Passiflora* showed the highest level of H-TAA between all the species studied in this research. On the other hand, for L-TAA, *Passiflora* and *Physalis* both had a higher balance of lipophilic antioxidants in the stems and roots, in comparison with *Solanum* (Figure 2). In this sense, the lowest levels of L-TAA were found in *Solanum*, regardless the part of the plant evaluated.

## 3. Discussion

### 3.1. Extraction Yield

Between the two extraction methods, the best yields were recorded by decoction, which gave averages of 18.35 ± 0.20% for *Passiflora*, 17.86 ± 0.16% for *Physalis* and 17.24 ± 0.20% for *Solanum*, versus 16.94 ± 0.94%, 17.81 ± 0.16% and 17.10 ± 0.19% (*Passiflora*, *Physalis* and *Solanum*, respectively) with maceration. These results were comparable to those obtained by Didi, A. [38] from *Arbutus unedo*, *Dapline gaidium* and *Cynara scolymus* [3] and Salem et al. [39] from *Nitraria retusa,* or Ozarowski et al. [40] from *Passiflora* species.

### 3.2. Total Polyphenol Content (TPC)

The TPC extracted by the decoction method was higher than that extracted by the maceration method for *Passiflora* and *Physalis*, and there were significant differences between the two methods of extraction. These results confirm that a moderate temperature would act in favor of the extraction; this fact has been confirmed by some authors who specify that the techniques using higher or lower temperatures and/or pressures would considerably increase the efficiency of the polyphenol extraction [39,41,42].

Small extraction yields could limit the use of some extracts of *Passiflora*, *Physalis* and *Solanum* (and plants in general) as sources of bioactive compounds. In addition, these compounds can sometimes be present in a very low concentration. On the other hand, and in consonance with Orlando et al. [43], we observed that the polyphenol extraction yields were considerably varied between different plant parts. In a recent study, and in agreement with our results, Salih et al. [44] did not obtain a good polyphenol extraction from macerated or decocted samples when using water as an extractant, in comparison with methanol and ethanol. These results were attributed to the lower polyphenol extraction yield obtained when water was used. However, the use of other extracting agents in these conventional techniques entails an optimal extraction of bioactive compounds [44]. In this sense, methanol’s polarity is higher than ethanol’s; for this reason, very polar phenolic acids, such as cinnamic or benzoic acids, could be extracted more easily, increasing the total polyphenol content in the extracts obtained [6]. This effect was in consonance with our results, since when higher methanol concentration were used, the TPC extraction became higher in comparison with methanol (70%) and the rest of the extractant agents for maceration or decoction in all plant species studied.

On the other hand, the work done by Martínez-Esplá et al. [45] on *Prunus avium* L. and Tomás-Barberán et al. [46] on nectarines, peaches and plums confirms our results by indicating that MeOH containing NaF allows for inactivated polyphenol oxidases, and prevents phenolic degradation due to browning. On the other hand, the high extraction potential of ethanol evaluated during decoction has also been observed by Katalinić et al. [47], Koffi et al. [48] and Mahmoudi et al. [3]. In these studies, ethanol in combination with water allowed for the better extraction of total polyphenols, because the addition of water to organic solvents increases the solubility of polyphenols and allows for maximum extraction [49].

### 3.3. Flavonoid Content

In these species studied, the majority of the secondary metabolites belong to flavonoids, and in particular, glycosides of the flavonols quercetin, rutine and myricetin [50,51,52]. In this study, and despite differences between the different parts of the plant, the leaves appear as the organ with the highest level of total flavonoids for all species studied. Leaves are always more exposed to sunlight than other plant organs. In fact, flavonoids protect plant tissues against the harmful effects of solar radiation [53]. The presence of flavonoids in the four organs of the plant could suggest that the plant has anti-inflammatory properties, and could thus play a positive role in the treatment of cardiovascular and neurodegenerative diseases, also perhaps having antitumor properties [54]. On the other hand, following our results, ethanolic solvents would be able to extract the highest amounts of total flavonoids from *Passiflora*, *Physalis* and *Solanum*. Mulinacci et al. [55] showed that an ethanol:water solution (7:3) increases the amount of flavonoids in the extraction. In this sense, when the aerial parts of *Passiflora caerulea* were evaluated using methanol, more flavonoid structures were detected (isoorientin, orientin, vitexin, saponarin and rutin) than when ethanol was used (isoorientin, orientin, vitexin, isovitexin) [56]. These findings could explain the higher level of total polyphenols and flavonoid content, which we found in our manuscript with methanol as an extractant agent in *Passiflora* showing the highest extractant polyphenol yield, followed by ethanol. However, ethanol and water are preferable because they have the advantage of being non-polluting, less expensive and non-toxic compared to other solvents, such as methanol [3,57]. The literature is scarce with respect to the plant parts of *Physalis peruviana.* However, in leaves, flavonoids such as quercetin, kaempferol and rutin were the most important secondary metabolites, while the main hydroxycinnamic acids detected were p-coumaric and caffeoyl-quinic acids [58]. Regarding *Solanum muricatum*, quercetin was the most important flavonoid, while ferulic and cinnamic acids were the most abundant phenolic acids detected [59].

### 3.4. Total Antioxidant Activity

The different plant parts of *Passiflora caerulea*, *Physalis peruviana* and *Solanum muricatum* Aiton are considered healthy because of their high levels of bioactive compounds, such as the phenolic compounds (total polyphenols, flavonoids), which contribute to the total antioxidant activity (TAA) [50,60,61]. The flowers and stems showed similar levels of H-TAA for *Passiflora*, reaching higher levels in its flowers as compared to all the values obtained in the rest of the flowers of this study. It is likely that the high levels of anthocyanins that *Passiflora* flowers contain could explain the significant differences between the flowers studied in this research [62,63].

The high levels of TAA in some plant parts and the low levels in other parts are related to their chemical compositions, functional groups of major compounds, and polyphenol content [54,64,65]. In fact, in *Physalis* the total polyphenol content in the leaves is twice as much as that in the flowers when applying most of the solvents studied in this research, especially with the decoction method. A similar pattern was observed in samples obtained after maceration, but the level of TPC extracted using this method was lower in the *Physalis* flowers, suggesting that maceration could extract the lower levels of these bioactive compounds located in the *Physalis* flowers (Table 2). Antioxidant activity generally depends on the number and position of the hydroxyl groups relative to the functional carboxyl groups [66]. In this sense, the TPC and flavonoid levels were in consonance with the TAA evaluated. Phenolic compounds are not the only contributors to the antioxidant activity in the water-soluble phase, since other compounds, such as ascorbic acid, show antioxidant activity in the water-soluble fraction. On the other hand, in the lipophilic fraction, different bioactive compounds, such as tocopherols, carotenoids and terpenes, also contribute to the total antioxidant activity of a particular sample [67]. In all the flowers evaluated, anthocyanins are present, conferring their characteristic purple color on some parts of this organ and contributing to the hydrophilic fraction with other phenolic compounds, such as isoorientin and orientin for *Passiflora*, or quercetin for *Physalis* and *Solanum*, especially in the leaves and roots. On the other hand, Salih et al. [44], in a different study, showed that lipophilic extracts were richer in tannins (mainly ellagic acid derivatives). In this study, the lipophilic fraction could also provide the antioxidant activity of other compounds, such as myristic acid for *Passiflora* [16] or β-carotene for *Physalis* [29] and *Solanum* [68].

In summary, these results show that in the three plants studied, the leaves and roots, as well as the flowers in the case of *Passiflora*, represent a very rich source of natural antioxidants.

## 4. Materials and Methods

### 4.1. Plant Material

Blue passion flower (*Passiflora caerulea* L.), cape gooseberry (*Physalis peruviana* L.) and pepino (*Solanum muricatum* Aiton) were harvested in September 2018 in Oran (Algeria). These plant species were harvested early in the morning with pruning shears and were tagged and transported in plastic bags. Leaves, flowers, stems and roots were separated, then dried at room temperature in darkness for 21 days and crushed using a grinder. Then samples were packaged in sealed polyethylene bags and transported to Spain at the University Miguel Hernández.

### 4.2. Chemical Products

The polyphenolic standards (gallic acid, hydrated quercetin-3-rutinoside), H_2_O_2_, ABTS peroxidase and trolox were provided by Sigma-Aldrich (Berlin, Germany). The reagents (Folin-Ciocalteu, aluminium trichloride, acetate of sodium) were provided by Sigma-Aldrich (Berlin, Germany), and the other solvents were obtained from Panreac (Barcelona, Spain), and Lab-Scan (Warsaw, Poland).

### 4.3. Extraction of Total Polyphenols and Flavonoids

Extraction by maceration—In order to extract the total polyphenols of the different plant parts by the maceration method described by Romani et al. [69], the following was carried out: 10 to 30 g of each of the crushed different parts (leaves, flowers, stems, roots) of the three plants were macerated for 2.5 h in triplicate at room temperature in 100 mL of aqueous solutions of the solvents (methanol, ethanol, acetone) diluted 7:3 in water, water and MeOH/water (8:2) containing 2 mM NaF according to Tomás-Barberán et al. [46]. After filtration through a muslin cloth, the filtrates were recovered and centrifuged at 4000 rpm at 4 °C for 20 min and then stored at −80 °C until use.

Extraction by decoction—In order to extract bioactive compounds by decoction, we performed the protocol described by Chavan et al. [70]. Quantities of 1 g of the powders of the different parts of the three plants were added to 40 mL of the different extraction solvents asmethanol, ethanol, acetone (diluted 7:3 in water) and MeOH/water (8:2), containing 2 mM NaF. Each mixture was boiled inside hermetically-sealed flasks in triplicate for 30 min in a water bath before it was filtered using a muslin cloth. The filtrates were recovered and centrifuged at 4000 rpm at 4 °C for 20 min and then stored at −80 °C until analysis of the extracted sample.

### 4.4. Extraction Yield

The yield of the two extraction methods for the three plants was calculated using the formula described by Falleh et al. [71]: Y (%) = 100 M_ext_/M_ech_, where Y is the yield of extraction in %, M_ext_ is the mass of the extract after the evaporation (using the rotary evaporator) of the extraction solvent in mg, and M_ech_ is the mass of the plant sample in mg.

### 4.5. Determination of Total Polyphenols and Flavonoids

Total polyphenols—Total polyphenols were determined by the Folin-Ciocalteu (FC) method [72,73]. In total, 200 μL of each extract was taken in duplicate to be analyzed and was added to 300 μL of 50 mM phosphate buffer solution, 2.5 mL of FC reagent and 2 mL of Na_2_CO_3_ at 1 N. The mixture was vortexed and then placed in a water bath at 50 °C for 5 min. The blank was performed by replacing the extract with the solution of phosphate buffer. The absorbance was measured at 760 nm using a UV-1700 Pharma spectrophotometer (Shimadzu). The results were expressed in mg gallic acid equivalent/g of dry vegetable material with reference to the calibration curve of gallic acid. All samples were analyzed in duplicate (*n* = 12).

Flavonoids—The flavonoid assay was performed according to the method detailed by Woisky and Salatino [74]. In total, 500 μL of each extract to be analyzed was mixed in duplicate with 1500 μL of methanol at 95%, 100 μL of AlCl_3_ at 10% (*m*/*v*), 100 μL of 1 M sodium acetate and 2.8 mL of distilled water. The whole mixture was vortexed and then incubated at room temperature in a dark condition for 30 min. The blank was made by replacing the extract with methanol at 95%, and the absorbance was measured at 415 nm by a UV-1700 Pharma spectrophotometer (Shimadzu). The results were expressed in mg equivalents of quercetin-3-rutinoside/100 g of dry matter with reference to the calibration curve of quercetin-3-rutinoside. All samples were analyzed in duplicate (*n* = 12). The selection of these methods allowed for comparison with other investigations, since they are the most common methods used to quantify these compounds with a good specificity and without additional equipment requirements.

### 4.6. Total Antioxidant Activity (TAA)

The determination of antiradical activity by the ABTS test was carried out using the method described by Cano et al. [75], slightly modified. In total, 10 mL of 50 mM phosphate buffer solution and 6 mL of ethyl acetate were added to 0.5 g of plant tissue (leaves, flowers, stems, and roots of the three plants) in three replicates and the mixture was homogenized for 1 min and then centrifuged at 10,000 rpm for 20 min at 4 °C. The hydrophilic and lipophilic phases of the different parts of the plants were separated.

Determination of the antioxidant activity in the hydrophilic phase (H-TAA)—In total, 890 μL of 50 mM glycine buffer solution was mixed with 30 μL of 10 mM ABTS solution, 30 μL of 1 mM H_2_O_2_ and 25 μL of 10 µM peroxidase. The absorbance of this mixture was measured at 730 nm against a blank prepared from the glycine solution buffer instead of a sample. In total, 25 μL of each water-soluble phase was added to the preceding mixture by duplicate (*n* = 6) and the absorbance was measured again at 730 nm after 1 min. The results were expressed in mg trolox equivalent/100 g of dry vegetable material with reference to the trolox calibration curve [75].

Determination of the antioxidant activity in the lipophilic phase (L-TAA)—In total, 30 μL of 10 mM ABTS solution was mixed with 30 μL of 1 mM H_2_O_2_, 25 μL of 10 µm peroxidase and 850 μL of ethanol. The absorbance of this mixture was measured at 730 nm against a blank prepared from ethanol. In total, 25 μL of each soluble lipid phase in duplicate (*n* = 6) was added to the preceding mixture and the absorbance was measured again at 730 nm after 1 min. The results were expressed in mg trolox equivalent/100 g of dry vegetable material with reference to the trolox calibration curve [75].

### 4.7. The Statistical Analyses

The treatments were distributed according to a complete randomized design (CRD). The data were analyzed via tree factors analysis of variance (methods, solvents and plant parts) procedures. Statistical analyses were performed with SAS software version 9.4 for Windows. Mean comparisons were performed using least significant difference (LSD) tests (*p* = 0.05) with standard deviation (SD) for the tables and standard error (SE) for the graphs.

## 5. Conclusions

The present work is the first study reporting information on these species of medicinal plants, with respect to the different plant parts. The extraction of total phenolic compounds and flavonoids is an important step in the evaluation of bioactive compounds and functional properties. The choice of the appropriate solvent and extraction technique is necessary in order to preserve the biological properties of these bioactive substances. From this work, decoction with methanol:water (8:2) containing 2 mM NaF was the preferred method of extraction of the total polyphenols. On the other hand, maceration with this solvent, and also with ethanol and acetone, were successful techniques for extracting flavonoids, especially from leaves (*Passiflora* and *Solanum*) and flowers for *Solanum*. This study reveals the difficulty of determining a single extraction process suitable for all different parts of the plant, showing significant differences between the three species studied. In all of them the results showed that the leaves and roots contain higher amounts of bioactive compounds, increasing their total antioxidant activity in comparison with other organs of the plant. In conclusion, with this research and regarding the medicinal possibilities of the different plant parts, the knowledge obtained increases the potential use of these medicinal plants in the prevention or treatment of many diseases affecting human health. In this sense, further investigation should be addressed elucidating the individual compounds with antioxidant properties that these species can provide.

## Figures and Tables

**Table 1:** Extraction yield (% DM) of the different parts of blue passion flower (*Passiflora caerulea* L.), cape gooseberry (*Physalis peruviana* L.) and pepino (*Solanum muricatum* Aiton) extracted by decoction and maceration.

**Extraction Method**	**Extraction Solvents**	**Blue Passion Flower (*Passiflora caerulea* L.)**			
		**Leaves**	**Flower**	**Stem**	**Roots**
Maceration	MeOH (70%)	15.44 ± 0.58 k–p	14.34 ± 1.33 m–q	12.07 ± 0.05 s	15.81 ± 0.4 l–p
	EtOH (70%)	13.22 ± 0.07 p–s	12.10 ± 2.14 p–s	12.83 ± 1.27 r,s	13.45 ± 2.7 n–r
	H_2_O	20.98 ± 0.27 a–c	18.73 ± 0.67 b–g	19.56 ± 7.43 g–n	18.81 ± 0.87 d–k
	Acetone (70%)	21.95 ± 0.05 a	20.32 ± 0.38 a–e	18.33 ± 0.53 c–i	21.07 ± 0.9 a–e
	MeOH, NaF (80%)	19.00 ± 0.95 d–j	15.28 ± 2.33 o–s	16.69 ± 1.67 j–p	18.70 ± 2.26 f–m
Decoction	MeOH (70%)	22.02 ± 0.02 a	20.02 ± 0.05 a–f	22.08 ± 0.96 a,b	22.18 ± 0.06 a
	EtOH (70%)	20.82 ± 0.17 a–d	18.42 ± 0.79 e–l	20.09 ± 0.27 a–f	20.53 ± 1.47 a–f
	H_2_O	18.87 ± 0.81 d–j	18.32 ± 1.63 f–m	16.68 ± 1.34 i–o	18.73 ± 0.54 b–g
	Acetone (70%)	13.73 ± 0.27 o–s	16.28 ± 0.07 h–o	12.25 ± 0.28 q–s	16.53 ± 1.93 l–p
	MeOH, NaF (80%)	19.07 ± 0.31 b–h	15.43 ± 1.11 i–o	16.83 ± 2.76 l–p	17.45 ± 0.1 f–n
**Extraction Method**	**Extraction Solvents**	**Cape Gooseberry (*Physalis peruviana* L.)**			
		**Leaves**	**Flower**	**Stem**	**Roots**
Maceration	MeOH (70%)	17.25 ± 0.14 l,m	15.02 ± 0.19 q	17.20 ± 0.16 l,m	18.76 ± 0.83 i–k
	EtOH (70%)	11.90 ± 0.67 s	16.02 ± 0.2 o,p	16.04 ± 0.21 o,p	12.81 ± 0.17 r
	H_2_O	18.87 ± 0.91 i–k	19.28 ± 0.39 g–i	17.76 ± 0.87 l,m	18.74 ± 0.83 i–k
	Acetone (70%)	20.33 ± 0.00 e,f	20.99 ± 1.25 e,f	20.90 ± 0.1 c–e	21.59 ± 0.29 a,b
	MeOH, NaF (80%)	16.96 ± 1.07 m–p	17.66 ± 0.82 l–n	18.93 ± 0.29 h–k	19.01 ± 0.23 g–j
Decoction	MeOH (70%)	19.53 ± 0.36 f,g	21.32 ± 0.35 a–c	20.31 ± 0.5 d,e	22.36 ± 0.13 a
	EtOH (70%)	17.22 ± 0.15 m,n	21.07 ± 0.14 b–e	19.38 ± 0.11 g,h	21.60 ± 0.64 b–d
	H_2_O	17.22 ± 0.3 l–n	17.90 ± 0.65 k,j	17.85 ± 0.33 k,l	18.23 ± 0.34 j,k
	Acetone (70%)	17.45 ± 0.88 m,n	13.06 ± 0.46 r	15.62 ± 0.25 p,q	16.41 ± 0.08 s
	MeOH, NaF (80%)	17.2 ± 0.73 m,n	17.09 ± 0.32 m–o	17.08 ± 0.16 m,n	16.20 ± 0.32 o,p
**Extraction Method**	**Extraction Solvents**	**Pepino (*Solanum muricatum* Aiton)**			
		**Leaves**	**Flower**	**Stem**	**Roots**
Maceration	MeOH (70%)	18.95 ± 0.17 e–g	15.78 ± 0.29 h–l	14.85 ± 0.55 o–q	15.79 ± 0.44 k–n
	EtOH (70%)	17.09 ± 0.36 h	15.44 ± 0.7 j–m	12.86 ± 0.33 r	14.28 ± 0.87 q
	H_2_O	18.63 ± 0.65 g	18.03 ± 0.35 g	16.17 ± 0.15 h–k	14.88 ± 0.43 n–q
	Acetone (70%)	21.41 ± 0.58 b	19.46 ± 0.4 d,e	19.34 ± 0.14 d–f	19.82 ± 0.01 d
	MeOH, NaF (80%)	19.54 ± 0.52 d–f	19.08 ± 0.44 d–f	16.05 ± 0.12 h–l	14.62 ± 0.83 m–p
Decoction	MeOH (70%)	22.67 ± 0.07 a	21.29 ± 0.01 b	19.63 ± 0.43 d–f	22.69 ± 0.1 a
	EtOH (70%)	20.98 ± 0.16 b	19.80 ± 0.89 e–f	14.97 ± 0.61 l–o	20.41 ± 0.68 c,d
	H_2_O	18.27 ± 0.54 f,g	18.18 ± 0.41 g	18.20 ± 2.02 h	18.61 ± 0.22 f,g
	Acetone (70%)	13.98 ± 0.59 p,q	14.43 ± 0.45 p,q	14.75 ± 0.67 o–p	12.57 ± 0.05 r
	MeOH, NaF (80%)	16.36 ± 0.71 h,i	16.09 ± 0.38 h–l	15.46 ± 0.3 l–o	16.64 ± 0.075 h–j

Different letters in the same column for each plant indicate significant differences at a *p* < 0.05 level of probability. Data are the mean ± standard deviation (SD).

**Table 2:** Total polyphenol content (mg eq GA/100 g DM) in the different parts of blue passion flower (*Passiflora caerulea* L.), cape gooseberry (*Physalis peruviana* L.) and pepino (*Solanum muricatum* Aiton) extracted by decoction and maceration.

**Extraction Method**	**Extraction Solvents**	**Blue Passion Flower (*Passiflora caerulea* L.)**			
		**Leaves**	**Flower**	**Stem**	**Roots**
Maceration	MeOH (70%)	1429.13 ± 55.62 g,h	1261.73 ± 47.89 j–m	1233.47 ± 74.25 l,m	1372.6 ± 60.81 h,i
	EtOH (70%)	1505.21 ± 65.65 c–e	1166.08 ± 47.89 n	1259.56 ± 75.26 k–m	1437.82 ± 67.12 f,g
	H_2_O	1324.78 ± 57.84 i,j	1083.47 ± 43.18 o	1198.69 ± 57.4 m,n	1137.82 ± 56.07 n,o
	Acetone (70%)	1476.95 ± 58.7 d–g	1274.78 ± 47.62 j–l	1290 ± 56.07 j–l	1316.08 ± 57.4 i–k
	MeOH, NaF (80%)	1976.95 ± 62.04 a	1498.69 ± 28.73 c–f	1518.26 ± 68.1 c,d	1466.08 ± 49.69 d–g
Decoction	MeOH (70%)	1234.13 ± 26.41 l,m	924.34 ± 24.33 r,s	974.34 ± 19.28 p–s	958.04 ± 24.17 p–s
	EtOH (70%)	1373.26 ± 32.02 h,i	964.56 ± 24.69 p–s	991.73 ± 15.26 p,q	1447.17 ± 19.24 e–g
	H_2_O	1229.78 ± 35.56 l,m	787.39 ± 28.51 t	918.91 ± 12.98 s	947.17 ± 28.92 q–s
	Acetone (70%)	1362.39 ± 47.41 i	938.47 ± 23.38 q–s	984.13 ± 9.64 p–r	1289.56 ± 31.15 j–l
	MeOH, NaF (80%)	1597.17 ± 41.45 b	1020 ± 27.26 p	997.17 ± 22.83 p,q	1551.52 ± 26.88 b,c
**Extraction Method**	**Extraction Solvents**	**Cape Gooseberry (*Physalis peruviana* L.)**			
		**Leaves**	**Flower**	**Stem**	**Roots**
Maceration	MeOH (70%)	1340 ± 36.54 f,g	940 ± 27.03 p	1050.86 ± 35.05 n	1576.95 ± 19.28 c
	EtOH (70%)	1774.78 ± 27.03 a	983.08 ± 30.81 o,p	1129.13 ± 37.14 m	1448.69 ± 29.27 d
	H_2_O	1205.21 ± 49.69 j,k	829.13 ± 29.59 q	1048.69 ± 30.94 n	1366.08 ± 32.14 e,f
	Acetone (70%)	1337.82 ± 31.25 f,g	968.26 ± 25.96 p	1220.43 ± 32.04 j,k	1492.17 ± 29.27 d
	MeOH, NaF (80%)	1635.65 ± 29.7 b	1016.08 ± 28.73 n,o	1296.52 ± 33.67 g,h	1637.82 ± 36.46 b
Decoction	MeOH (70%)	1141.73 ± 27.72 l,m	480.86 ± 9.39 t	1120 ± 37.73 m	1224.34 ± 29.05 i,j
	EtOH (70%)	1221.08 ± 40.53 j,k	809.13 ± 29.38 q,r	1177.6 ± 37.96 k,l	1268.91 ± 24.94 h,i
	H_2_O	1026.52 ± 31.25 n,o	422.17 ± 18.27 u	772.17 ± 31.15 r	1099.34 ± 28.7 m
	Acetone (70%)	1237.39 ± 43.91 I,j	490.65 ± 20.81 t	1134.13 ± 41.91 l,m	1348.26 ± 24.72 f
	MeOH, NaF (80%)	1393.91 ± 39.37 e	558.04 ± 31.22 s	1291.73 ± 25.96 h	1554.78 ± 37.06 c
**Extraction Method**	**Extraction Solvents**	**Pepino (*Solanum muricatum* Aiton)**			
		**Leaves**	**Flower**	**Stem**	**Roots**
Maceration	MeOH (70%)	1266.08 ± 44.33 e	818.26 ± 27.03 r	887.826 ± 36.54 q	1159.56 ± 37.82 g,h
	EtOH (70%)	1335.65 ± 48.67 d	1024.78 ± 29.59 j–l	1131.3 ± 40.47 h	1168.26 ± 30.43 g,h
	H_2_O	1163.91 ± 46.76 g,h	716.08 ± 35.05 s	840 ± 37.56 r	966.08 ± 32.14 n–p
	Acetone (70%)	1340 ± 45.73 d	892.17 ± 31.75 q	1016.08 ± 36.46 k–m	1026.95 ± 27.03 j–l
	MeOH, NaF (80%)	1637.82 ± 47.29 b	1013.91 ± 31.75 l,m	1172.6 ± 37.82 g,h	1398.69 ± 36.46 c
Decoction	MeOH (70%)	1198.26 ± 36.89 f,g	921.08 ± 24.94 p,q	963.47 ± 23.81 n–p	1045 ± 34.48 i–k
	EtOH (70%)	1403.69 ± 22.83 c	950.43 ± 29.05 o,p	1002.6 ± 24.84 i–k	1061.3 ± 33.01 i–k
	H_2_O	1074.34 ± 22.02 i	920 ± 31.75 p,q	738.47 ± 35.03 s	900.43 ± 25.72 q
	Acetone (70%)	1374.34 ± 27.61 c,d	1009.13 ± 25.22 l–n	977.6 ± 23.1 m–o	1045 ± 33.93 i–k
	MeOH, NaF (80%)	1824.34 ± 27.72 a	1068.91 ± 27.81 i,j	1268.91 ± 21.41 e	1243.91 ± 27.15 e,f

Different letters in the same column for each plant indicate significant difference at a *p* < 0.05 level of probability. Data are the mean ± standard deviation (SD).

**Table 3:** Flavonoid content (mg eq Qu/100 g DM) in the different parts of blue passion flower (*Passiflora caerulea* L.), cape gooseberry (*Physalis peruviana* L.) and pepino (*Solanum muricatum* Aiton) extracted by decoction and maceration.

**Extraction Method**	**Extraction Solvents**	**Blue passion flower (*Passiflora caerulea* L.)**			
		**Leaves**	**Flower**	**Stem**	**Roots**
Maceration	MeOH (70%)	532.71 ± 5.28 c	162.84 ± 1.05 s–u	222.46 ± 4.91 m–p	200.48 ± 0.35 r,q
	EtOH (70%)	676 ± 9.82 b	222.46 ± 10.52 m–p	242.49 ± 1.2 k–m	239.03 ± 4.21 k–m
	H_2_O	329.92 ± 1.01 e	100.6 ± 1.4 w	132.6 ± 0.7 v	129.44 ± 0.35 v
	Acetone (70%)	717.28 ± 1.4 a	217.48 ± 2.45 n–q	265.57 ± 8.23 h–j	237.82 ± 0.7 k–n
	MeOH, NaF (80%)	736.1 ± 5.38 a	166.78 ± 0.7 s–u	154.94 ± 0.35 t,u	233.27 ± 0.35 l–o
Decoction	MeOH (70%)	300.42 ± 19.63 f,g	173.89 ± 0.7 s,t	183.6 ± 0.7 r,s	236.42 ± 1.4 k–n
	EtOH (70%)	318.99 ± 5.61 e,f	301.63 ± 4.21 f	218.82 ± 0.7 n-q	472.85 ± 5.48 d
	H_2_O	211.53 ± 3.51 o–q	198.78 ± 1.4 qr	158.71 ± 1.4 tu	147.17 ± 2.1 u,v
	Acetone (70%)	297.74 ± 2.1 fg	246.74 ± 0.7 j-l	255.24 ± 0.7 k-n	238.24 ± 0.7 k–n
	MeOH, NaF (80%)	273.74 ± 5.6 hi	203.03 ± 3.51 p-r	278.56 ± 6.83 gh	229.14 ± 2.8 l–o
**Extraction Method**	**Extraction Solvents**	**Cape Gooseberry (*Physalis peruviana* L.)**			
		**Leaves**	**Flower**	**Stem**	**Roots**
Maceration	MeOH (70%)	205.34 ± 3.86 k	49 ± 1.4 t	115.78 ± 2.1 q	219.6 ± 0.7 g–j
	EtOH (70%)	257.55 ± 2.19 e	55.68 ± 3.51 s,t	148.57 ± 4.91 n,o	282.14 ± 2.8 c
	H_2_O	116.09 ± 3.86 q	23.8 ± 0.35 u	73.59 ± 1.05 r	133.39 ± 0.7 p
	Acetone (70%)	277.59 ± 1.77 c	63.26 ± 3.15 r,s	136.43 ± 5.61 o,p	300.66 ± 8.58 b
	MeOH, NaF (80%)	230.53 ± 7.71 f,g	45.66 ± 1.75 t	155.55 ± 10.17 n	279.11 ± 2.1 c
Decoction	MeOH (70%)	209.1 ± 1.93 k,j	57.31 ± 2.1 s,t	215.78 ± 7.01 h–k	279.53 ± 3.51 c
	EtOH (70%)	323.85 ± 2.64 a	155.07 ± 1.2 n	217.6 ± 13.32 h–k	272.24 ± 4.72 c,d
	H_2_O	169.64 ± 9.82 m	140.49 ± 1.4 o,p	170.24 ± 2.1 g–k	210.32 ± 0.7 i–k
	Acetone (70%)	234.6 ± 2.1 e	175.71 ± 7.01 l	222.46 ± 4.91 f–i	315.35 ± 2.62 a
	MeOH, NaF (80%)	260.1 ± 9.11 d,e	157.49 ± 2.8 m,n	224.28 ± 4.21 f–h	252.82 ± 3.51 e
**Extraction Method**	**Extraction Solvents**	**Pepino (*Solanum muricatum* Aiton)**			
		**Leaves**	**Flower**	**Stem**	**Roots**
Maceration	MeOH (70%)	402.17 ± 3.51 e	120.46 ± 6.31 s,t	97.22 ± 2.29 v–x	171.34 ± 0.35 p,q
	EtOH (70%)	649.89 ± 7.71 a	128.96 ± 6.12 r,s	158.71 ± 1.63 q	229.02 ± 0.35 i–k
	H_2_O	224.89 ± 4.72 j–l	67.03 ± 2.1 y	112.56 ± 8.41 t,u	137.64 ± 1.4 r
	Acetone (70%)	550.32 ± 3.32 b	127.74 ± 3.51 r,s	187.24 ± 2.1 n,o	236.6 ± 0.7 h–j
	MeOH, NaF (80%)	440.42 ± 2.04 d	84.03 ± 0.7 x	99.81 ± 2.1 u–w	202 ± 4.21 m,n
Decoction	MeOH (70%)	252.82 ± 2.33 g	41.53 ± 0.7 y	159.92 ± 1.40 q	190.89 ± 3.51 n,o
	EtOH (70%)	502.96 ± 3.55 c	49.42 ± 4.21 y	178.14 ± 4.21 o,p	214.57 ± 7.01 k–m
	H_2_O	212.74 ± 0.7 l,m	17.85 ± 1.4 z	87.67 ± 0.7 w,x	227.92 ± 5.61 i–k
	Acetone (70%)	277.71 ± 4.21 f	61.87 ± 2.56 y	159.92 ± 2.8 q	241.28 ± 2.8 g–i
	MeOH, NaF (80%)	288.64 ± 1.4 f	52.15 ± 2.22 y	107.71 ± 2.8 t–v	246.74 ± 0.7 g–h

Different letters in the same column for each plant indicate significant differences at a *p* < 0.05 level of probability. Data are the mean ± standard deviation (SD).
